# The Interaction between Root Herbivory and Competitive Ability of Native and Invasive-Range Populations of *Brassica nigra*

**DOI:** 10.1371/journal.pone.0141857

**Published:** 2015-10-30

**Authors:** Ayub M. O. Oduor, Marc Stift, Mark van Kleunen

**Affiliations:** Ecology, Department of Biology, University of Konstanz. Universitätsstrasse 10, D-78457 Konstanz, Germany; Helmholtz Centre for Environmental Research (UFZ), GERMANY

## Abstract

The evolution of increased competitive ability (EICA) hypothesis predicts that escape from intense herbivore damage may enable invasive plants to evolve higher competitive ability in the invasive range. Below-ground root herbivory can have a strong impact on plant performance, and invasive plants often compete with multiple species simultaneously, but experimental approaches in which EICA predictions are tested with root herbivores and in a community setting are rare. Here, we used *Brassica nigra* plants from eight invasive- and seven native-range populations to test whether the invasive-range plants have evolved increased competitive ability when competing with *Achillea millefolium* and with a community (both with and without *A*. *millefolium*). Further, we tested whether competitive interactions depend on root herbivory on *B*. *nigra* by the specialist *Delia radicum*. Without the community, competition with *A*. *millefolium* reduced biomass of invasive- but not of native-range *B*. *nigra*. With the community, invasive-range *B*. *nigra* suffered less than native-range *B*. *nigra*. Although the overall effect of root herbivory was not significant, it reduced the negative effect of the presence of the community. The community produced significantly less biomass when competing with *B*. *nigra*, irrespective of the range of origin, and independent of the presence of *A*. *millefolium*. Taken together, these results offer no clear support for the EICA hypothesis. While native-range *B*. *nigra* plants appear to be better in dealing with a single competitor, the invasive-range plants appear to be better in dealing with a more realistic multi-species community. Possibly, this ability of tolerating multiple competitors simultaneously has contributed to the invasion success of *B*. *nigra* in North America.

## Introduction

The evolution of increased competitive ability (EICA) hypothesis predicts that escape from intense herbivory and subsequent genetically based re-allocation of resources to increased growth and reproductive output may help invasive plant species to colonize novel habitats in the invasive range [1]. The EICA hypothesis is derived from the optimal defense hypothesis assuming that defenses are costly because they divert resources from growth and reproduction [1,2]. Accordingly, the EICA hypothesis predicts that plants from the invasive range that evolved lower anti-herbivore defenses should evolve increased competitive ability. Hence, when grown under common conditions in the absence of herbivores, invasive-range plants should exhibit significantly higher growth and reproductive output than conspecific plants from the native range [1]. In the presence of herbivores, however, the competitive advantage of plants from the invasive range should be reduced or reversed [1,2]. In most of the experiments testing predictions of the EICA hypothesis, invasive-range and conspecific native-range plants were grown under common conditions in the absence of competitors [3]. In the few studies where competition was explicitly included, only intraspecific competition [4] or pairwise interspecific competitive interactions between plants from invasive and native ranges versus their neighbors were investigated [3,5–7]. However, invasive plants typically compete in multi-species plant communities that may vary with regard to the presence of strong competitor species [8–12]. Therefore, tests for evolution of increased competitive ability in invasive plants require manipulative experiments that compare the performance of invasive-range and conspecific native-range plants under competition in a community setting.

Because plant roots perform such vital functions as uptake of water and nutrients necessary for plant growth [13], herbivore damage on plant roots can cause significant reductions in individual plant growth and fitness, and can influence overall plant community structure [14,15]. Hence, escape from root herbivory may play an important role in explaining increased competitive ability in invasive-range plants. However, of the few studies that have tested the effect of herbivory on competitive ability of invasive-range and conspecific native-range plants [3,5,16], only one tested for the effects of root herbivory. The study found that invasive-range *Chromolaena odorata* plants outcompeted native-range *C*. *odorata* plants, both in the presence and absence of root herbivory [16]. However, as this study tested the effect of root herbivory only in the presence of intraspecific competition, the effect of root herbivory on competitive ability of invasive-range and conspecific native-range plants in a community setting remains unknown.

Within natural plant communities, competitive interactions between plants have two components: competitive effect (i.e., the degree to which a focal plant suppresses the growth of its neighbors) and competitive response (i.e., the degree to which a focal plant can tolerate the impact of its neighbors) [17–20]. In plant invasions, these two components of competition may characterize different invasion stages; competitive response would be an important determinant of establishment success of the invader within a recipient community, whereas the competitive effect would be important for impact of the invader to the community [6]. However, the few experiments that have investigated both competitive effects and responses of invasive-range and conspecific native- range plants have focused only on pairwise competitive interactions [6,7,21,22], and found mixed results.

In this study, we tested for the separate and joint effects of specialist root herbivory on *B*. *nigra* and the presence of a community of competitors on the growth of *B*. *nigra* plants from the invasive and native ranges, and the competitive effect of *B*. *nigra* on the community. We specifically addressed the following questions: i) In the absence of root herbivory, do *B*. *nigra* plants from the invasive range have a higher competitive ability (i.e., a weaker biomass reduction due to growth in a community of competitors and a stronger negative effect on the community) than *B*. *nigra* plants from the native range?; ii) Do *B*. *nigra* plants from the invasive range suffer more from root herbivory than *B*. *nigra* plants from the native range?; and iii) Does root herbivory change the competitive responses and effects of *B*. *nigra* plants from the invasive and native ranges differently?

## Materials and Methods

### Study species and seed sources

*Brassica nigra* (Brassicaceae) (L.) W. D. J. Koch is a self-incompatible annual herb native to Europe, Asia and North Africa, and was introduced to North America approximately 200 years ago [23–25]. Seeds of *B*. *nigra* have long been used in southern Europe, Asia and North Africa for cooking oil, condiment mustard and medicine [25], and most likely for this reason the species has been introduced to other continents. Presently, *B*. *nigra* is invasive in certain regions of North America, where it can form thick monospecific stands [26]. Recent studies have found significantly higher resistance to generalist herbivory (in line with the shifting defence hypothesis) [27,28] and higher reproductive output in the invasive-range populations of *B*. *nigra* relative to their native-range conspecifics, suggesting rapid post-introduction evolution of the invasive-range populations of *B*. *nigra* [5,29].

As competitors of *B*. *nigra*, we selected five plant species (two grasses and three forbs). To ensure that native- and invasive-range *B*. *nigra* plants had a comparable history of co-evolution with the competitor species, we selected competitor species that co-occur with *B*. *nigra* in large parts of both its native and invasive range (The Jepson Interchange, information: http://ucjeps.berkeley.edu/interchange/). The grasses were *Elymus glaucus* and *Nasella pulchra*, and the forbs were *Medicago lupulina*, *Sonchus oleraceus* and *Achillea millefolium*. While we did not have *a priori* information on the competitive effects of the first four species, *A*. *millefolium* is known to exert strong competitive effects on other plant species that are invasive in the same range as *B*. *nigra* [30–32]. Bulked samples of seeds collected from several maternal plants in eight invasive-range and seven native-range populations of *B*. *nigra* as well as the five competitor species were obtained directly from the field or from seed germplasm collections (see S1 and S2 Tables).

### Ethics statement

Permission to import seeds from North America was granted by the German ministry of agriculture and food (permit number: SAG-2013-018-AG-7, dated 1^st^ July 2013). The imported seeds had been collected from field sites where the plants grow naturally, and no specific permission was required to access those sites as they were unprotected public land. Furthermore, none of the species used in the present study is endangered or under official protection. As the present study was conducted in the greenhouse, no specific permission was required for that purpose.

### Pre-cultivation and experimental design

To test whether *B*. *nigra* plants from the invasive and native ranges differed in their responses to growth in a community setting, and whether these responses depended on damage by a specialist root herbivore of *B*. *nigra*, we performed a greenhouse experiment in the botanical garden of the University of Konstanz (Germany) between September 2013 and January 2014. In September 2013, seeds of *B*. *nigra* and the five competitor species were sown individually in plastic plug-trays filled with a commercial potting soil (Standard soil, Gebr. Patzer GmbH & Co. KG, Sinntal, Germany; organic matter content: 40–50%, pH (H_2_O): 4.5–7.0, electrical conductivity: 200–900μS/cm). The trays were kept in a phytochamber (12h day/night cycle at 21°C/17°C and 90% relative humidity). In a previous germination trial, *B*. *nigra* seeds had germinated five days earlier than the five competitor species, and hence for the experiment, the competitors were sown a week earlier (on 23^rd^ September 2013) than *B*. *nigra* seeds. After three weeks, we transplanted the emerged seedlings to 2.5-L round plastic pots filled with sand and vermiculite mixed in a ratio of 1:1. In each pot, we applied 10 g of a slow-release fertilizer (Osmocote Classic 14% N, 14% P_2_O_5_, 14% K_2_O; Scotts, Geldermalsen, The Netherlands).

Employing a full factorial design, we grew individual *B*. *nigra* plants from invasive- and native-range populations in the presence or absence of a community of the four plant species without *a priori* information on the strength of their competitive effects (*E*. *glaucus*, *N*. *pulchra*, *M*. *lupulina* and *S*. *oleraceus*). This factor was crossed with the presence or absence of a species that *a priori* was known to exert strong competitive effects (*A*. *millefolium*) [30–32]. In treatments where *B*. *nigra* was grown with four or five competitors (five or six plants per pot, respectively), an individual *B*. *nigra* plant always occupied a central position in the pot while the other competitor species were distributed randomly to one of four or five positions at equal distances around the *B*. *nigra* plant. In the treatment where an individual *B*. *nigra* plant was grown in pairwise competition with an individual *A*. *millefolium* (two plants per pot), the plants were planted on opposite ends of a pot. In the no competition treatment, a single *B*. *nigra* plant was grown in the center of the pot. Although an effect of adding *A*. *millefolium* might mainly be due to a further increase in density of the competitors, this experimental design nevertheless allows us to test whether the effect of the increase in density differs for native- and invasive-range *B*. *nigra* plants. The factorial combination of the four competition treatments (no competition, competition with the single strong competitor *A*. *millefolium*, competition with the community in the absence of a strong competitor *A*. *millefolium* and competition with the community in the presence of a strong competitor *A*. *millefolium*) were further crossed with a root herbivory treatment (presence vs. absence of root herbivory on *B*. *nigra* plants).

We imposed the root herbivory treatments on *B*. *nigra* plants three and a half weeks after transplanting. As a herbivore, we took *Delia radicum* (L.) (Diptera: Anthomyiidae), a specialist herbivore exclusively feeding on Brassicaceae and native to Europe [33]. Although *D*. *radicum* has been introduced to the north-eastern coast of North America (Newfoundland, Canada) in the 19^th^ century, where it has been reported as a serious pest of Brassicaceous crops [34,35], no report exists of the insect attacking wild populations of invasive-range *B*. *nigra*. The invasive-range populations of *B*. *nigra* that we used in the present study were, therefore, considered to have escaped damage by *D*. *radicum*. One half of all the *B*. *nigra* plants in the experiment were infested with *D*. *radicum* by placing five eggs around the root collar of the *B*. *nigra* plant using a fine-tipped paint brush. The root collar is also the typical location where females of *D*. *radicum* lay their eggs [33]. The eggs were slightly covered with moist sand to prevent desiccation. Emerged larvae burrow into the soil to feed on roots until they pupate [33]. To confirm that the larvae indeed fed on the roots in our experiment, we grew an additional set of 12 *B*. *nigra* test plants; six individual plants with eggs and six individual plants without eggs. Five weeks after placing the eggs, we uprooted the 12 test plants for visual inspection of root herbivore damage. All six infested plants showed clear damage, and the control plants were undamaged (S1 Fig). At the time of harvest, visual observation again confirmed clear *D*. *radicum* damage on the experimentally infested *B*. *nigra* plants and no damage on the control plants.

As the number of populations constitutes the effective number of replicates for testing differences between the native and invasive ranges, we maximized the number of populations (15) over the number of replicates (4 per treatment) per population [36].Thus, for each of the 15 *B*. *nigra* populations (eight from the invasive range and seven from the native range), we had eight treatment combinations (2 x 2 x 2: presence/absence of community x presence/absence of *A*. *millefolium* x presence/absence of root herbivory on *B*. *nigra*), which resulted in 480 (i.e., 4 x 8 x 15) experimental pots. In addition, we grew eight replicates of: i) the community (*E*. *glaucus*, *N*. *pulchra*, *M*. *lupulina*, and *S*. *oleraceus*) without competition from *B*. *nigra* and *A*. *millefolium*, and ii) the community in competition with *A*. *millefolium*. These additional treatments served as controls for testing the suppressive effects of *B*. *nigra* and *A*. *millefolium* on the community. This resulted in an additional 16 pots. The 496 experimental pots were assigned to four separate blocks in two greenhouse compartments (two blocks per compartment) employing a complete randomized block design whereby each treatment and population combination appeared in every block. The pots were spaced 0.35 m apart. The greenhouse conditions were maintained at a temperature regime of 24 ± 5°C, a light cycle of 16 h: 8 h (Day/Night), and 50–70% relative humidity. The plants were watered once a day by filling plastic plates placed beneath each pot with tap water.

### Measurement of biomass yield

We used aboveground biomass as proxy for fitness since aboveground biomass and seed yield of annual plant species are often positively correlated [37]. Previous experiments had confirmed this for *B*. *nigra* [5,29]. We did not harvest below-ground biomass, because it was impossible to separate roots of different species in the competition treatments. All experimental plants were harvested after three months of growth. This was done by cutting the individual plants at the root collar and then placing all the above-ground material belonging to an individual plant in separate paper bags. The individual plants were then dried at 70°C for 72 hours, and then weighed.

### Statistical analysis

To test whether *B*. *nigra* plants from the invasive and native ranges differed in their biomass responses to the different competition treatments, root herbivory and their interactions, we used linear mixed-effects models fitted with the *lme* function in the R package *nlme* [38]. The fixed part of the model included four effects, each with two levels: Achillea (presence vs. absence of *A*. *millefolium*), community (presence vs. absence), herbivory treatment (presence vs. absence of root herbivory on *B*. *nigra* plants), *B*. *nigra* range (invasive vs. native) and all possible interactions, reflecting the full factorial design of our experiment. Maternal resource provisioning to developing seeds can influence early acting plant traits such as seedling growth [39]. To avoid potential bias due to such maternal effects, we used initial height and total leaf count of four-week old individual plants as co-variates in the models. The random part of the model included population (nested in *B*. *nigra* range) and block. The model accounted for heteroscedasticity among populations using the *varIdent* function available within the *lme* function [40].

A principal components analysis indicated that changes in community biomass were driven by unidirectional responses of all the four community members (i.e., the first principal component that explained 41% variance had positive loadings for each of the individual community members, S3 Table). Therefore, our analyses of the competitive effect of *B*. *nigra* on the community focused on the total community biomass (rather than separately analyzing the biomass of individual species). Because we obviously could not grow the community with the *B*. *nigra* specialist herbivore when *B*. nigra was absent, we did not have a full factorial design for community biomass. Therefore, we did separate analyses to test for effects of *B*. *nigra* and herbivory on community biomass. First, to test the general effects of presence/absence of *B*. *nigra* and of *A*. *millefolium* on community productivity, we selected the subset of cases without herbivory (n = 136), and then constructed linear mixed-effects models. The model fixed parts included Brassica (presence vs. absence of *B*. *nigra*), *B*. *nigra* range (invasive vs. native; fitted sequentially after *B*. *nigra* presence), Achillea (presence vs. absence of *A*. *millefolium*) and all possible interactions; the random part included block. Second, to test whether root herbivory on *B*. *nigra* mediated the competitive effect of *B*. *nigra* on the community productivity, we selected the subset of cases in which the community was grown with *B*. *nigra* plants (n = 240), and constructed linear mixed-effects models. The model fixed part included *B*. *nigra* range (invasive vs. native), root herbivory on *B*. *nigra* (herbivory vs. no herbivory), Achillea (presence vs. absence of *A*. *millefolium*) and all possible two and three-way interactions; the random part included block and *B*. *nigra* population (nested in range).

For all models, we tested the significance of the interactions and main effects by removing first, the highest order interactions, and then the lower order interactions and finally the main effects, and performing model comparisons using likelihood-ratio tests (see notes below Tables 1–3 for the exact comparisons). All analyses were performed in R v3.0.3 [41].

**Table 1:** Results of likelihood-ratio model comparisons of nested linear mixed models to test whether *B*. *nigra* range (invasive vs. native), community (presence vs. absence of *Elymus glaucus*, *Nasella pulchra*, *Medicago lupulina* and *Sonchus oleraceus*), Achillea (presence vs. absence of *Achillea millefolium*), root herbivory on *B*. *nigra* (herbivory vs. no herbivory), and their interactions had a significant effect on aboveground biomass yield of *B*. *nigra*. Significant factors are marked in bold.

Effect	*χ* ^2^(df = 1)	*P*
Number of leaves at four weeks^a^	0.054	0.814
Height at four weeks^a^	35.55	**< 0.0001**
Range (R)^b^	0.004	0.950
Achillea (A)^b^	0.73	0.390
Community (C)^b^	30.64	**< 0.0001**
Herbivory (H)^b^	0.001	0.971
R x A^c^	1.19	0.274
R x C^c^	1.27	0.259
R x H^c^	3.33	0.067
A x C^c^	8.04	**0.004**
A x H^c^	0.84	0.359
C x H^c^	5.99	**0.014**
R x A x C^d^	5.99	**0.014**
R x A x H^d^	< 0.001	0.994
R x C x H^d^	0.48	0.487
A x C x H^d^	0.06	0.807
R x A x C x H^e^	0.03	0.874

Initial height and number of leaves of a month-old *B*. *nigra* plants were included in the models as co-variates. Populations and blocks were included in the models as random effects, and heteroscedastocity among populations was accounted for by calculating separate variances for each population using the VarIdent function.

^a^ Removal of effect compared to model without fixed part.

^b^ Removal of effect compared to: covariates + random part + Range (R) + Achillea (A) + Community (C) + Herbivory (H).

^c^ Removal of effect compared to: covariates + random part + Range (R) + Achillea (A) + Community (C) + Herbivory (H) + 2-way interactions between R, A, C and H.

^d^ Removal of effect compared to: covariates + random part + Range (R) + Achillea (A) + Community (C) + Herbivory (H) + 2-way and 3-way interactions between R, A, C and H.

^e^ Removal of effect compared to: covariates + random part + Range (R) + Achillea (A) + Community (C) + Herbivory (H) + 2-way, 3-way and 4-way interactions between R, A, C and H.

**Table 2:** Results of likelihood-ratio model comparisons of nested linear mixed-effects models to test whether *B*. *nigra* (presence vs. absence), *B*. *nigra* range (invasive vs. native; fitted sequentially after *B*. *nigra*), Achillea presence (presence vs. absence of *Achillea millefolium*), and all possible interactions had a significant effect on the community aboveground biomass. Significant factors are marked in bold.

Effect	*χ* ^2^(df = 1)	*P*
*Brassica nigra* (presence/absence)^a^	11.53	**0.0007**
*Achillea millefolium* (presence/absence)^a^	1.43	0.231
*B*. *nigra* range (invasive/native)^b^	1.83	0.176
*B*. *nigra* x *A*. *millefolium* ^c^	0.55	0.459
*B*. *nigra* range x *A*. *millefolium* ^d^	0.21	0.644

^a^Removal of effect compared to: random part + *B*.*nigra* + *A*. *millefolium*.

^b^Removal of effect compared to: random part + *A*. *millefolium* + *B*. nigra + *B*. *nigra* range.

^c^Removal of effect compared to: random part + *A*. *millefolium* + *B*. *nigra* + *B*. *nigra* range + *A*. *millefolium* x *B*. *nigra*.

^d^Removal of effect compared to: random part + *A*. *millefolium* + *B*. *nigra* + *B*. *nigra* range + *A*. *millefolium* x *B*. nigra + *A*. *millefolium* x *B*. *nigra* Range.

**Table 3:** Results of likelihood-ratio model comparisons of nested linear mixed models to test whether *B*. *nigra range* (invasive vs. native), herbivory on *B*. *nigra* (herbivory vs. no herbivory), *A*. *millefolium* presence (presence vs. absence), and all possible two and three-way interactions had a significant effect on the community aboveground biomass.

Effect	*χ* ^2^(df = 1)	*P*
Range^a^	0.46	0.498
Herbivory^a^	1.21	0.272
*Achillea millefolium* ^a^	0.35	0.554
Range x Herbivory^b^	1.76	0.185
*A*. *millefolium* x Herbivory^b^	0.76	0.381
Range x *A*. *millefolium* ^b^	2.53	0.111
Herbivory x Range x *A*. *millefolium* ^c^	0.74	0.391

^a^Removal of effect compared to: random part + Herbivory + Range + *A*. *millefolium*.

^b^Removal of effect compared to: random part + Herbivory + Range + *A*. *millefolium* + 2-way interactions between Range, Herbivory, and *A*. *millefolium*.

^c^Removal of effect compared to: random part + Herbivory + Range + *A*. *millefolium* + 2-way and 3-way interactions between Range, Herbivory, and *A*. *millefolium*.

## Results

### *Brassica nigra* biomass

**Fig 1:**
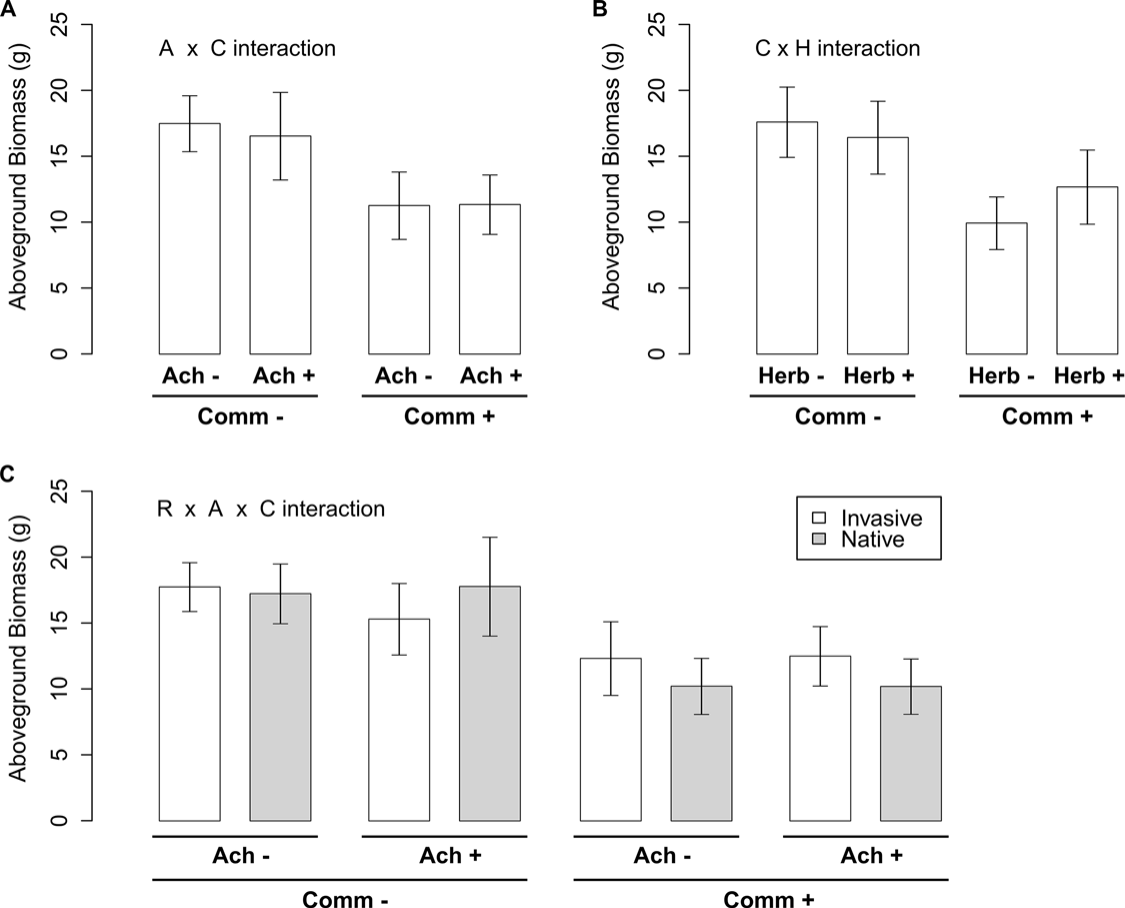
Mean (± 1SE) *Brassica nigra* biomass illustrating a) A x C: the 2-way interaction between *Achillea millefolium* (A: presence/absence) and community (C: presence/absence of *Elymus glaucus*, *Nasella pulchra*, *Medicago lupulina* and *Sonchus oleraceus*); b) C x H: the 2-way interaction between C and herbivory on *B*. *nigra* (H: presence/absence); c) R x A x C: the 3-way interaction between *B*. *nigra* range (R: invasive/native) and A and C. The means and standard error (SE) were calculated as follows: 1) for each combination of factor levels, we calculated the mean and standard deviation of population means; 2) for each interaction plot, we calculated the mean of the factor level means that were not involved in the plotted interaction, and standard errors based on the mean standard deviations and the sample size of the smallest group (n = 7).

On average, competition with the community significantly reduced aboveground biomass of *B*. *nigra* by 33.0% (Table 1). The presence of *A*. *millefolium* had only a small negative effect on biomass of *B*. *nigra*, and only so in the absence of the community (-5.99% in the absence of a community *vs* + 0.73% in presence of the community; significant Achillea x Community (A x C) interaction: Table 1; Fig 1A). In the absence of the community, biomass of invasive-range *B*. *nigra* plants was reduced by the presence of *A*. *millefolium* (-13.9%; Fig 1C), while this was not the case for native-range *B*. *nigra* plants (+ 0.7%; Fig 1C). On the other hand, invasive-range *B*. *nigra* plants suffered less from the presence of the community (-24.6%) than native-range *B*. *nigra* plants (-40.6%), regardless of the presence/absence of *A*. *millefolium*. This was reflected by a significant Range x Achillea x Community (R x A x C) three-way interaction (Table 1; Fig 1C).

Although the overall effect of herbivory on biomass of *B*. *nigra* was not significant, it reduced the negative effect of the presence of the community (-42.7% *vs* -22.7%; significant Community x Herbivory (C x H) interaction: Table 1; Fig 1B). Four-way and all the other three-way and two-way interactions were not significant (Table 1; S2 Fig). Nevertheless, there was a marginally significant interactive effect of herbivory and *B*. *nigra* range on *B*. *nigra* biomass; while *B*. *nigra* plants from the invasive range suffered from herbivory (-8.1%), native -range *B*. *nigra* plants produced more biomass under herbivory (+18.3%) irrespective of presence or absence of competitors (Table 1; S2C Fig).

### Community biomass

**Fig 2:**
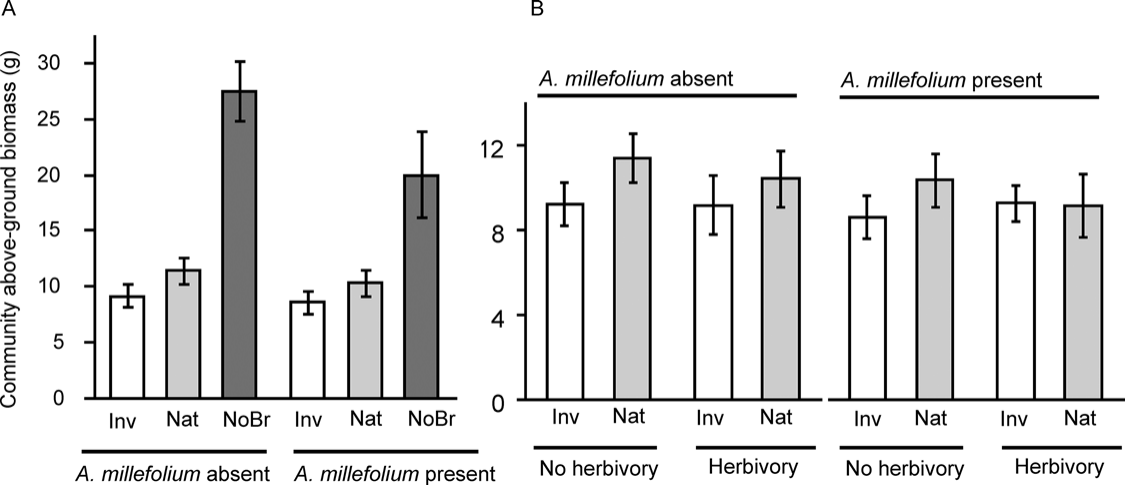
Mean (± 1SE) above-ground biomass of a community of four species (*Elymus glaucus*, *Nasella pulchra*, *Medicago lupulina* and *Sonchus oleraceus*) grown in the: a) absence (NoBr) versus presence of *B*.*nigra* plants from the invasive (Inv) or native (Nat)-range crossed with absence versus presence of *Achillea millefolium*, b) absence versus presence of root herbivory on invasive- or native-range *B*.*nigra* plants crossed with absence versus presence of *A*.*millefolium*.

The community produced significantly less aboveground biomass when grown in the presence of *B*. *nigra* than in the absence of *B*. *nigra* (Table 2; Fig 2A). Range of *B*. *nigra*, root herbivory on *B*. *nigra*, presence of *A*. *millefolium* or any interaction between these factors did not significantly affect community biomass (Table 3; Fig 2B).

## Discussion

The evolution of increased competitive ability (EICA) hypothesis predicts that in the absence of herbivore damage, plants from the invasive range should exhibit higher competitive ability than conspecific plants from the native range [1]. Despite intensive research on the EICA hypothesis [42–44], the effect of herbivory on post-introduction evolution of competitive ability in invasive plants remains little studied to date [3]. In this paper, we show that native-range *B*. *nigra* plants competed better than invasive-range *B*. *nigra* plants in the presence of a single strong competitor *A*. *millefolium*, while invasive-range *B*. *nigra* plants were better competitors than native-range *B*. *nigra* plants in a more realistic multi-species community setting. Irrespective of the presence of competitors, invasive-range *B*. *nigra* plants tended to suffer from root herbivory, whereas native-range *B*. *nigra* plants benefitted from it, although there was only a marginally significant interaction between *B*. *nigra* range and herbivory. Overall, these results offer no clear support for the EICA hypothesis. While native-range *B*. *nigra* plants appear to be better in dealing with a single competitor, the invasive-range plants appear to be better in dealing with a more realistic multi-species community. Possibly, this ability of tolerating multiple competitors simultaneously has contributed to the invasion success of *B*. *nigra* in North America.

### Competitive ability of *B*. *nigra* plants from the invasive and native ranges

The significant three-way interaction between range of *B*. *nigra*, presence of *A*. *millefolium* and presence of community (R x A x C in Table 1) partly supports and partly contradicts the EICA hypothesis. On the one hand, regardless of whether *A*. *millefolium* was part of the community, invasive-range *B*. *nigra* plants suffered less from the presence of the community than did native-range *B*. *nigra* plants (Fig 1C), which supports a prediction of the EICA hypothesis. On the other hand, native-range *B*. *nigra* plants suffered less when grown in pairwise competition with *A*. *millefolium* in the absence of community than did the invasive-range *B*. *nigra* plants (Fig 1C), which is contrary to a prediction of the EICA hypothesis. The finding that invasive-range and native-range *B*. *nigra* plants had similar competitive effects on the community regardless of root herbivory treatment on *B*. *nigra* is also contrary to a prediction of the EICA hypothesis (Fig 2B). Most support for the EICA hypothesis stems from studies that have confirmed the prediction of significantly higher growth in invasive- relative to native-range conspecifics, for example in *Barbarea vulgaris*, *Cardaria draba*, *Rorippa austriaca* and *Jacobaea vulgaris* [45,46]. However, this patterns is not universal, as similar vegetative growth and reproduction was reported for invasive- and native-range plants of *Mimulus guttatus* [47] and *Lythrum salicaria* [48]. Only few other studies have examined the prediction of increased competitive ability by manipulating competitive environments, and these also produced mixed results. Similar competitive responses were reported for invasive- and native-range plants of *Eschscholzia californica* [49], *Silene latifolia* [50], and *Lepidium draba* [51]. In contrast, higher competitive response (i.e., higher biomass production) was reported for invasive-range *Sapium sebiferum* plants than their native-range conspecifics [52]. On the other hand, invasive-range *Alliaria petiolata* had a significantly lower competitive response (i.e., lower biomass production) than their native-range conspecifics [4]. However, because all these previous studies reported only the competitive responses of conspecific plants from the native and invasive-ranges, and no results on the competitive effects, they provide incomplete picture regarding the possible post-introduction evolution of overall competitive ability in invasive plant species.

Our study adds to the few other experiments that have investigated both competitive effects and responses of invasive-range and native- range plants on other plant species [6,7,21,22]. Invasive-range *Lythrum salicaria* plants exhibited significantly stronger competitive effects and responses than *L*. *salicaria* plants from the native range when both groups of plants were grown in pairwise intraspecific and interspecific competition [6]. Invasive-range plants of *Centaurea maculosa* exhibited stronger competitive effects and responses than native-range *C*. *maculosa* plants when grown in pairwise competitive interactions with *Pseudoroegneria spicata* or *Festuca idahoensis* [21]. In another study, invasive- and native-range plants of *C*. *maculosa* had similar competitive effects and responses [22]. In contrast, grown in pairwise interspecific competition with *Urtica dioica*, plants of *Impatiens glandulifera* from the invasive range exhibited weaker competitive effects and were more suppressed by their neighbors relative to native-range *I*. *glandulifera* plants [7]. Clearly, there is no universal pattern emerging from the studies focusing only on pairwise competitive interactions, and we, therefore, assessed competitive effects and responses in a community setting.

Our experimental design provided some indication that, although native-range *B*. *nigra* performed better in a pairwise interaction with *A*. *millefolium*, invasive-range *B*. *nigra* plants may be better competitors than native-range *B*. *nigra* plants in a multi-species community setting. This suggests that invasive-range *B*. *nigra* plants evolved mechanisms that allow them to tolerate strong competition. Numerous comparative studies have shown that invasive plant species exhibit higher mean values of traits that contribute to competitive ability than native and non-invasive plant species, including a higher capacity to acquire and retain growth resources and/or to exploit resources better (e.g., through early growth and plastic morphological responses such as root-foraging responses) than co-occurring native species [53,54]. It remains to be tested whether such traits explain why invasive-range *B*. *nigra* plants appear to be better in dealing with a more realistic multi-species community.

### Effects of root herbivory on competitive ability of *B*. *nigra*

One of our main findings is that root herbivory by a Brassicaceae specialist reduced the negative effects of competition on *B*. *nigra* grown in a community setting (Fig 1B). This seems counterintuitive as the specialist root herbivore only targets Brassicaceae, and not the community. A possible explanation is that attack by the root herbivore induced compensatory growth in *B*. *nigra*. Indeed, plants can exhibit increased competitive ability when damaged by herbivores through compensatory growth [21,55–57]. Our finding that the beneficial effect of root herbivory tended to be stronger (although only marginally so) in *B*. *nigra* from the native range (Table 1 & S2C Fig) suggests that the native-range *B*. *nigra* plants have higher compensatory growth (i.e., higher tolerance of herbivory) than invasive- range *B*. *nigra*. This confirms previous field experiments with *B*. *nigra* that found that the native-range populations expressed significantly higher levels of compensatory growth than the invasive-range populations following damage by a community of above-ground feeding herbivores [5,29].

This pattern may be the result of differential herbivore selection pressures in the respective ranges, and thus have resulted from post-introduction selection imposed by herbivores. Invasive *B*. *nigra*, like many other invasive species [28,58], has not escaped herbivory, but has experienced a change in the level of herbivory and the community of herbivores it interacts with. Specifically, invasive-range *B*. *nigra* interacts more with generalist and less with specialist herbivore species than native-range counterparts [5]. Phylogeographic evidence indicates that invasive-range populations of *B*. *nigra* were introduced from multiple sources in the native range [59], which not only may have facilitated the invasion due to admixture boosting fitness [60], but also have provided standing genetic variation that natural selection can act upon [61,62]. This suggests that invasive-range populations of *B*. *nigra* should have sufficient standing genetic variation for selection to operate on.

Shifts in interactions with herbivores may thus have selected for the lower tolerance in response to root herbivory that we observed in invasive-range *B*. *nigra* plants compared to native-range *B*. *nigra* plants (marginally significant R x H interaction, Table 1; S2C Fig), which weakly supports the shifting defense hypothesis. The shifting defence hypothesis predicts that biogeographical differences in the levels of herbivory and composition of the herbivore community may select for high resistance (expression of high concentrations of less costly qualitative defence compounds like glucosinolates that are most effective against generalist herbivores) and low tolerance (compensatory growth that is most beneficial to plants that are attacked by a high diversity and density of both generalist and specialist herbivores) [27,63]. In a study similar to ours, genotypes of the North American invader *C*. *maculosa* that expressed higher compensatory growth in response to herbivore damage (i.e., higher tolerance) also demonstrated stronger competitive responses than genotypes of *C*. *maculosa* with lower compensatory growth [21]. However, more studies with similar setups are needed to more conclusively test the shifting defense hypothesis, and the prediction that selection for traits associated with increased tolerance (increased rates of photosynthesis and resource acquisition, and ultimately growth) may indirectly select for increased competitive ability [21,55–57].

## Supporting Information

S1 Fig *Roots of individual Brassica nigra plants* damaged (a) or undamaged (b) by larvae of a specialist root herbivore (*Delia radicum*). Note the black lesions caused by larval feeding on the damaged root.
(TIF)

S2 Fig Mean (± 1SE) *Brassica nigra* biomass for a) R x A: the 2-way interaction between *B*.*nigra* range (R: Inv = invasive; Nat = native) and *Achillea millefolium* (A: absence = Ach-; presence = Ach+); b) R x C: the 2-way interaction between R and Community (C: absence = Comm-; presence = Comm+ of *Elymus glaucus*, *Nasella pulchra*, *Medicago lupulina* and *Sonchus oleraceus*); c) R x H: the 2-way interaction between R and herbivory on *B*. *nigra* (H: absence = Herb-; presence = Herb+); d) A x H: the 2-way interaction between A and H. The means and standard error (SE) were calculated as follows: 1) for each combination of factor levels, we calculated the mean and standard deviation of population means; 2) for each interaction plot, we calculated the mean of the factor level means that were not involved in the plotted interaction, and standard errors based on the mean standard deviations and the sample size (number of populations) of the smallest group (n = 7).
(TIF)

S1 File Data used to test whether *B*. *nigra* plants from the invasive and native ranges differed in their biomass responses to the different competition treatments, root herbivory on *B*. *nigra* and their interactions. (CSV)

S2 File Data used to analyze for the competitive effect of *B*. *nigra* on the community biomass yield. (XLS)

S1 Table *Brassica nigra* seed sources for the current experiment. Populations marked by † were obtained from the United States Department of Agriculture (USDA) GRIN germplasm collections. Seeds for the French population were obtained from Leibniz Institute of Plant Genetics and Crop Plant Research (IPK)—Germany. Asterisks (*) indicate populations whose exact collection sites were not provided by GRIN germplasm collections.
(DOC)

S2 Table Seed sources of five competitor species used in the current experiment. Species marked by † were obtained from United States Department of Agriculture (USDA) GRIN germplasm collections.
(DOC)

S3 Table Correlations (loadings) of the original variables (biomass of each community member) to the four principal components (PC1-PC4). (DOC)

## References

[1] Blossey B, Nötzold R. Evolution of increased competitive ability in invasive non-indigenous plants: a hypothesis. J Ecol. 1995; 83: 887–889. 10.2307/2261425

[2] Herms DA, Mattson WJ. The dilemma of plants: to grow or defend. Q Rev Biol. 1992;67: 283–335. 10.1086/417659

[3] Felker-Quinn E, Schweitzer JA, Bailey JK. Meta-analysis reveals evolution in invasive plant species but little support for evolution of increased competitive ability (EICA). Ecol Evol. 2013; 3: 739–751. 10.1002/ece3.488 23531703 PMC3605860

[4] Bossdorf O, Prati D, Auge H, Schmid B. Reduced competitive ability in an invasive plant. Ecol Lett. 2004; 7: 346–353. 10.1111/j.1461-0248.2004.00583.x

[5] Oduor AMO, Strauss SY, Garcia Y, Cascales MB, Gomez JM. Herbivores mediate different competitive and facilitative responses of native and invader populations of *Brassica nigra* . Ecology. 2013; 94: 2288–2298. 10.1890/12-2021.1 24358714

[6] Joshi S, Gruntman M, Bilton M, Seifan M, Tielbörger K. A comprehensive test of evolutionarily increased competitive ability in a highly invasive plant species. Ann Bot. 2014; 114 (8): 1761–1768. 10.1093/aob/mcu199 25301818 PMC4649698

[7] Gruntman M, Pehl AK, Joshi S, Tielbörger K. Competitive dominance of the invasive plant *Impatiens glandulifera*: using competitive effect and response with a vigorous neighbour. Biol Invasions. 2013; 16:141–151. 10.1007/s10530-013-0509-9

[8] Callaway RM, Aschehoug ET. Invasive plants versus their new and old neighbors : a mechanism for exotic invasion. Science. 2000; 290: 521–523. 10.1126/science.290.5491.521 11039934

[9] Catford JA, Jansson R, Nilsson C. Reducing redundancy in invasion ecology by integrating hypotheses into a single theoretical framework. Divers Distrib. 2009;15: 22–40. 10.1111/j.1472-4642.2008.00521.x

[10] Heard MJ, Sax DF. Coexistence between native and exotic species is facilitated by asymmetries in competitive ability and susceptibility to herbivores. Ecol Lett. 2013; 16: 206–213. 10.1111/ele.12030 23157598

[11] Metlen KL, Callaway RM. Native North American pine attenuates the competitive effects of a European invader on native grasses. Biol Invasions. 2014; 17: 1227–1237 10.1007/s10530-014-0790-2

[12] Oduor AMO. Evolutionary responses of native plant species to invasive plants: a review. New Phytol. 2013; 200: 986–992. 10.1111/nph.12429 24712050

[13] Bazzaz FA, Chiariello NR, Coley PD, Pitelka LF. Allocating resources to reproduction and defense. Bioscience. 1987; 37: 58–67. 10.2307/1310178

[14] Bezemer TM, van Dam NM. Linking aboveground and belowground interactions via induced plant defenses. Trends Ecol Evol. 2005; 20: 617–624. 10.1016/j.tree.2005.08.006 16701445

[15] Zvereva EL, Kozlov MV. Sources of variation in plant responses to belowground insect herbivory: a meta-analysis. Oecologia. 2012; 169: 441–452. 10.1007/s00442-011-2210-y 22159919

[16] Zheng Y, Feng Y, Zhang L, Callaway RM, Valiente-Banuet A, Luo D, et al Integrating novel chemical weapons and evolutionarily increased competitive ability in success of a tropical invader. New Phytol. 2015; 205: 1350–1359. 10.1111/nph.13135 25367824

[17] Goldberg DE, Fleetwood L. Competitive effect and response in four annual plants. J Ecol. 1987; 75: 1131–1143.

[18] Goldberg DE, Landa K. Competitive effect and responses: hierarchies and correlated traits in the early stages of competition. J Ecol. 1991; 79: 1013–1030.

[19] Keddy PA, Twolan-Strutt L, Wisheu IC. Competitive effect and response rankings in 20 wetland plants: are they consistent across three environments? J Ecol. 1994; 82: 635–643. 10.2307/2261270

[20] Cahill JF, Kembel SW, Gustafson DJ. Differential genetic influences on competitive effect and response in *Arabidopsis thaliana* . J Ecol. 2005; 93: 958–967. 10.1111/j.1365-2745.2005.01013.x

[21] Ridenour W, Vivanco J, Feng Y, Horiuchi J, Callaway R. No evidence for trade-offs: *Centaurea* plants from America are better competitors and defenders. Ecol Monogr. 2008; 78: 369–386. 10.1890/06-1926.1

[22] He W-M, Feng Y, Ridenour WM, Thelen GC, Pollock JL, Diaconu A, et al Novel weapons and invasion: biogeographic differences in the competitive effects of *Centaurea maculosa* and its root exudate (+/-)-catechin. Oecologia. 2009; 159: 803–815. 10.1007/s00442-008-1234-4 19219462

[23] Bell DT, Muller CH. Dominance of California annual grasslands by *Brassica nigra* . Am Midl Nat. 1973; 90: 277–299. 10.2307/2424453

[24] Feeny P, Rosenberry L. Seasonal variation in the glucosinolate content of North American *Brassica nigra* and *Dentaria* species. Biochem Syst Ecol. 1982; 10: 23–32. 10.1016/0305-1978(82)90047-3

[25] Westman AL, Kresovich S. Simple sequence repeat (SSR) -based marker variation in *Brassica nigra* genebank accessions and weed populations. Euphytica. 1999; 109: 85–92. 10.1023/A:1003637814963

[26] Lankau RA, Strauss SY. Community complexity drives patterns of natural selection on a chemical defense of *Brassica nigra* . Am Nat. 2008;171: 150–161. 10.1086/524959 18197768

[27] Doorduin LJ, Vrieling K. A review of the phytochemical support for the shifting defence hypothesis. Phytochem Rev. 2011; 10: 99–106. 10.1007/s11101-010-9195-8 21475397 PMC3047680

[28] Müller-Schärer H, Schaffner U, Steinger T. Evolution in invasive plants: implications for biological control. Trends Ecol Evol. 2004;19: 417–422. 10.1016/j.tree.2004.05.010 16701299

[29] Oduor AMO, Lankau RA, Strauss SY, Gómez JM. Introduced *Brassica nigra* populations exhibit greater growth and herbivore resistance but less tolerance than native populations in the native range. New Phytol. 2011; 191: 536–544. 10.1111/j.1469-8137.2011.03685.x 21410474

[30] Bourdȏt GW, Saville DJ, Field RJ. The response of *Achillea millefolium* L. (yarrow) to shading. New Phytol. 1984; 97: 653–663. 10.1111/j.1469-8137.1984.tb03629.x

[31] Pickering C, Hill W. Roadside weeds of the snowy mountains, Australia. Mt Res Dev. 2007;27: 359–367. 10.1659/mrd.0805

[32] Deck A, Muir A, Strauss SY. Transgenerational soil-mediated differences between plants experienced or naïve to a grass invasion. Ecol Evol. 2013;3: 3663–3671. 10.1002/ece3.716 24198931 PMC3810866

[33] van Dam NM, Samudrala D, Harren FJM, Cristescu SM. Real-time analysis of sulfur-containing volatiles in Brassica plants infested with root-feeding *Delia radicum* larvae using proton-transfer reaction mass spectrometry. AoB Plants. 2012;pls021 10.1093/aobpla/pls021 22916330 PMC3424660

[34] Dixon PL, Coady JR, Larson DJ, Spaner D. Undersowing rutabaga with white clover: impact on *Delia radicum* (Diptera: Anthomyiidae) and its natural enemies. Can Entomol. 2004;136: 427–442. 10.4039/n03-067

[35] Biron DG, Nenon JP, Coderre D, Boivin G. Intra- and inter-specific variations on the chorionic ultrastructures of Delia eggs (Diptera: Anthomyiidae). Ann Entomol Soc Am. 2003;96: 245–249. 10.1603/0013-8746

[36] van Kleunen M, Dawson W, Bossdorf O, Fischer M. The more the merrier: multi-species experiments in ecology. Basic Appl Ecol. 2014;15: 1–9. 10.1016/j.baae.2013.10.006

[37] Shipley B, Dion J. The allometry of seed production in herbaceous angiosperms. Am Nat. 1992;139: 467–483.

[38] Pinheiro J, Bates D, DebRoy S, Sarkar D, RC team. nlme: linear and nonlinear mixed effects models [Internet]. 2007. Available: http://cran.r-project.org/web/packages/nlme/index.html

[39] Ouborg NJ, van Treuren R. Variation in fitness-related characters among small and large populations of *Salvia pratensis* . J Ecol. 1995;83: 369–380. 10.2307/2261591

[40] Zuur A, Ieno E, Walker N, Saveliev A, Smith G. Mixed effects models and extensions in ecology with R Springer, New York; 2009.

[41] R Development Core Team. A language and environment for statistical computing R Foundation for Statistical Computing, Vienna (2013).

[42] Bossdorf O, Auge H, Lafuma L, Rogers WE, Siemann E, Prati D. Phenotypic and genetic differentiation between native and introduced plant populations. Oecologia. 2005;144: 1–11. 10.1007/s00442-005-0070-z 15891837

[43] Colautti RI, Ricciardi A, Grigorovich IA, MacIsaac HJ. Is invasion success explained by the enemy release hypothesis? Ecol Lett. 2004;7: 721–733. 10.1111/j.1461-0248.2004.00616.x

[44] Joshi J, Vrieling K. The enemy release and EICA hypothesis revisited: incorporating the fundamental difference between specialist and generalist herbivores. Ecol Lett. 2005;8: 704–714. 10.1111/j.1461-0248.2005.00769.x

[45] Buschmann H, Edwards PJ, Dietz H. Variation in growth pattern and response to slug damage among native and invasive provenances of four perennial Brassicaceae species. J Ecol. 2005;93: 322–334. 10.1111/j.1365-2745.2005.00991.x

[46] Stastny M, Schaffner U, Elle E. Do vigour of introduced populations and escape from specialist herbivores contribute to invasiveness? J Ecol. 2005;93: 27–37. 10.1111/j.1365-2745.2004.00962.x

[47] van Kleunen M, Fischer M. Adaptive rather than non-adaptive evolution of *Mimulus guttatus* in its invasive range. Basic Appl Ecol. 2008;9: 213–223. 10.1016/j.baae.2007.03.006

[48] Willis AJ, Thomas MB, Lawton JH. Is the increased vigour of invasive weeds explained by a trade-off between growth and herbivore resistance? Oecologia. 1999;120: 632–640. 10.1007/s004420050899 28308315

[49] Leger EA, Rice KJ. Invasive California poppies (*Eschscholzia californica* Cham.) grow larger than native individuals under reduced competition. Ecol Lett. 2003;6: 257–264. 10.1046/j.1461-0248.2003.00423.x

[50] Blair AC, Wolfe LM. The evolution of an invasive plant: an experimental study with *Silene latifolia* . Ecology. 2004;85: 3035–3042. 10.1890/04-0341

[51] McKenney JL, Cripps MG, Price WJ, Hinz HL, Schwarzländer M. No difference in competitive ability between invasive North American and native European *Lepidium draba* populations. Plant Ecol. 2007;193: 293–303. 10.1007/s11258-007-9268-y

[52] Zou J, Rogers WE, Siemann E. Increased competitive ability and herbivory tolerance in the invasive plant *Sapium sebiferum* . Biol Invasions. 2007;10: 291–302. 10.1007/s10530-007-9130-0

[53] van Kleunen M, Weber E, Fischer M. A meta-analysis of trait differences between invasive and non-invasive plant species. Ecol Lett. 2010;13: 235–245. 10.1111/j.1461-0248.2009.01418.x 20002494

[54] Keser LH, Visser EJW, Dawson W, Song Y-B, Yu F-H, Fischer M, et al Herbaceous plant species invading natural areas tend to have stronger adaptive root foraging than other naturalized species. Front Plant Sci. 2015;6: 1–9. 10.3389/fpls.2015.00273 25964790 PMC4410514

[55] Callaway RM, De Luca TH. Biological-control hebivores may increase competitive ability of the noxious weed *Centauria maculosa* . Ecology. 1999;80: 196–1201. 10.1890/0012-9658

[56] Siemens DH, Lischke H, Maggiulli N, Schürch S, Roy BA. Cost of resistance and tolerance under competition: the defense-stress benefit hypothesis. Evol Ecol. 2003;17: 247–263. 10.1023/A:1025517229934

[57] Jones T, Kulseth S, Mechtenberg K, Jorgenson C, Zehfus M, Brown P, et al Simultaneous evolution of competitiveness and defense: induced switching in *Arabis drummondii* . Plant Ecol. 2006;184: 245–257. 10.1007/s11258-005-9070-7

[58] Liu H, Stiling P. Testing the enemy release hypothesis: a review and meta-analysis. Biol Invasions. 2006;8: 1535–1545. 10.1007/s10530-005-5845-y

[59] Oduor AMO, Gomez JM, Herrador MB, Perfectti F. Invasion of *Brassica nigra* in North America:distributions and origins of chloroplast DNA haplotypes suggest multiple introductions. Biol Invasions. 2015;17: 2447–2459. 10.1007/s10530-015-0888-1

[60] van Kleunen M, Röckle M, Stift M. Admixture between native and invasive populations may increase invasiveness of *Mimulus guttatus* . Proc R Soc B. 2015;282 10.1098/rspb.2015.1487 PMC4614753 26354937

[61] Dlugosch KM, Parker IM. Founding events in species invasions: genetic variation, adaptive evolution, and the role of multiple introductions. Mol Ecol. 2008;17: 431–449. 10.1111/j.1365-294X.2007.03538.x 17908213

[62] Rius M, Darling JA. How important is intraspecific genetic admixture to the success of colonising populations? Trends Ecol Evol. 2014;29: 233–242. 10.1016/j.tree.2014.02.003 24636862

[63] Müller-Schärer H, Schaffner U, Steinger T. Evolution in invasive plants: implications for biological control. Trends Ecol Evol. 2004;19: 417–422. 10.1016/j.tree.2004.05.010 16701299

