# Supplementary figures and images for: The Interaction between Root Herbivory and Competitive Ability of Native and Invasive-Range Populations of Brassica nigra
Source: PLoS One. 2015 Oct 30;10(10):e0141857. doi: 10.1371/journal.pone.0141857 (PMC4627727; doi:10.1371/journal.pone.0141857)

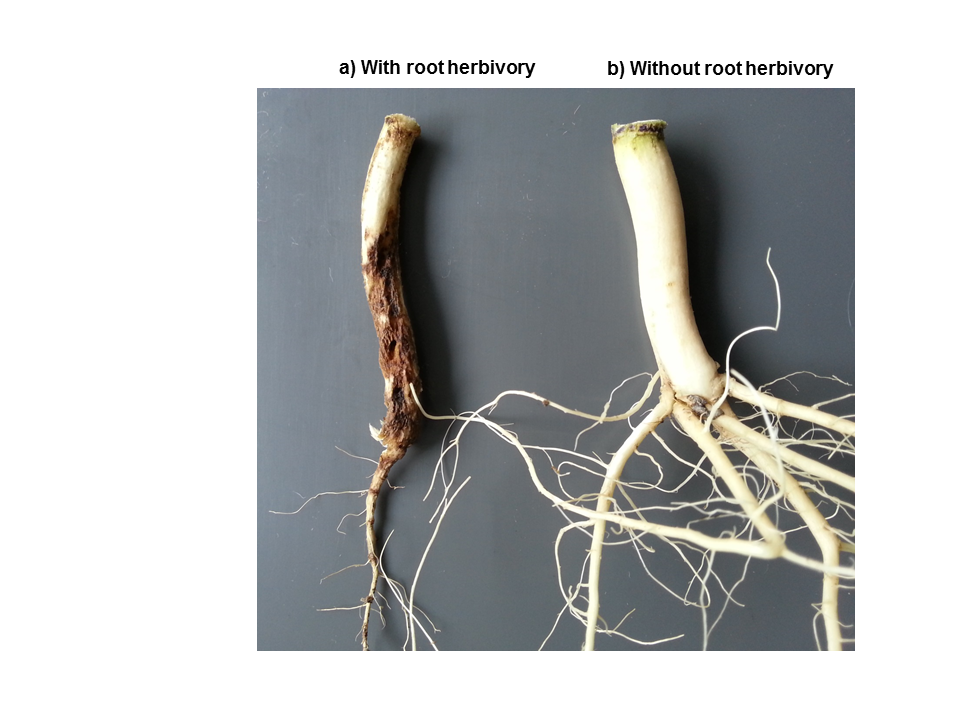

Supplement: S1 Fig — Note the black lesions caused by larval feeding on the damaged root. (TIF) [file pone.0141857.s001.tif]

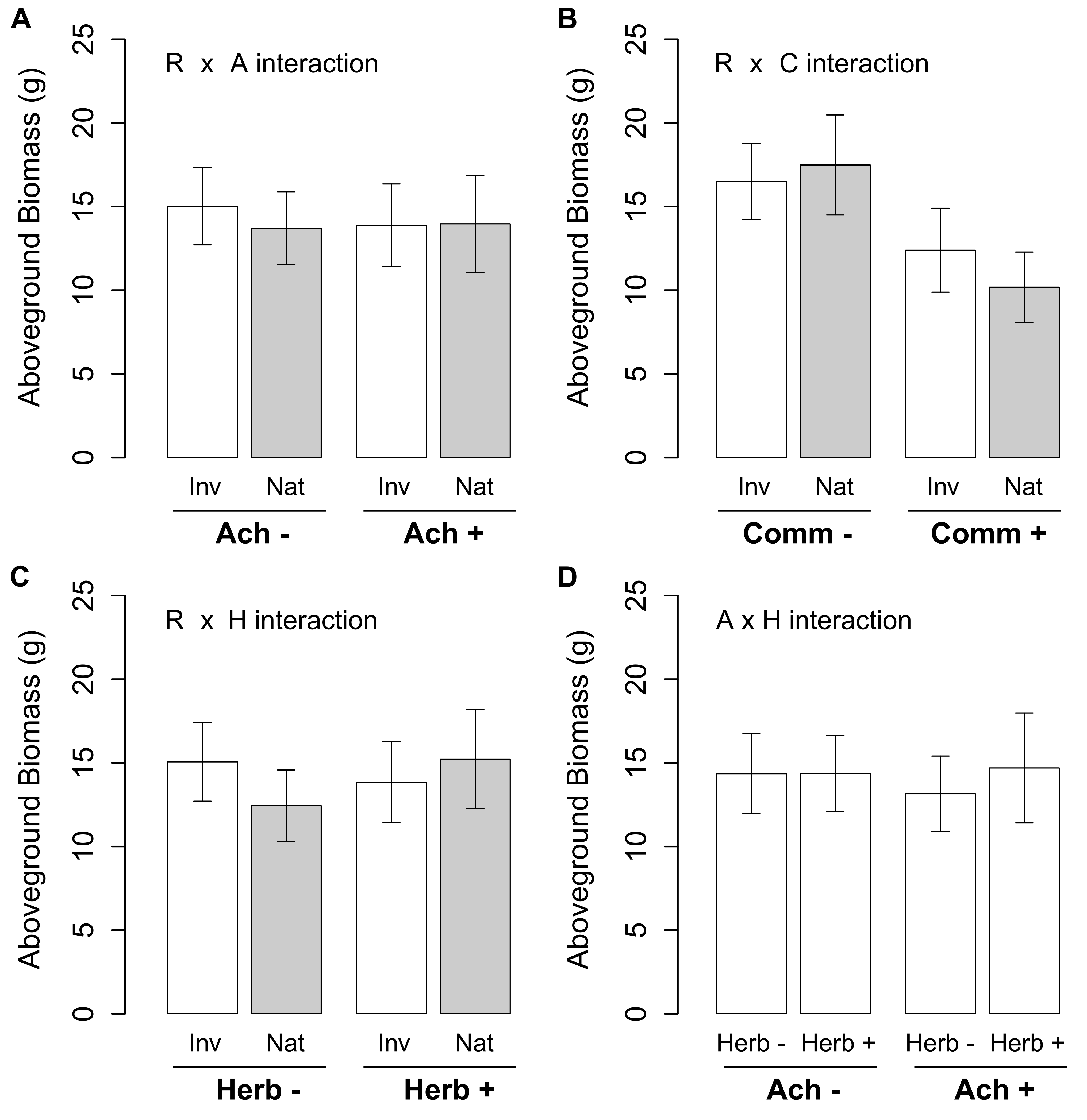

Supplement: S2 Fig — The means and standard error (SE) were calculated as follows: 1) for each combination of factor levels, we calculated the mean and standard deviation of population means; 2) for each interaction plot, we calculated the mean of the factor level means that were not involved in the plotted interaction, and standard errors based on the mean standard deviations and the sample size (number of populations) of the smallest group (n = 7). (TIF) [file pone.0141857.s002.tif]
