# Supplementary material for: The Interaction between Root Herbivory and Competitive Ability of Native and Invasive-Range Populations of Brassica nigra
Source: PLoS One. 2015 Oct 30;10(10):e0141857. doi: 10.1371/journal.pone.0141857 (PMC4627727; doi:10.1371/journal.pone.0141857)
Supplement: S1 Table — Populations marked by † were obtained from the United States Department of Agriculture (USDA) GRIN germplasm collections. Seeds for the French population were obtained from Leibniz Institute of Plant Genetics and Crop Plant Research (IPK)—Germany. Asterisks (*) indicate populations whose exact collection sites were not provided by GRIN germplasm collections. (DOC) [file pone.0141857.s005.doc]

# S1 Table

| Population | Country/State of seed collection | Accession number  or collector’s name | Geographic region | Invasive status |
| --- | --- | --- | --- | --- |
| South Elk Grove | California, USA. | S.Y. Strauss | North America | Invasive |
| Napa county | California, USA. | R. Lankau | North America | Invasive |
| UC Davis airport (Yolo County) | California, USA. | S.Y. Strauss | North America | Invasive |
| Ithaca | New York, USA | J. Conner | North America | Invasive |
| Banfield | Michigan, USA | J. Lau | North America | Invasive |
| Ontario† | Canada | PI649154 | North America | Invasive |
| Champaign | Illinois, USA | J. Conner | North America | Invasive |
| Urbana | Illinois, USA | J. Conner | North America | Invasive |
| *† | Ethiopia | PI597830 | Africa | Native |
| *† | Poland | PI 358590 | Central Europe | Native |
| Borneo | Spain | Lidia Caño | Mediterranean | Native |
| Afferden | The Netherlands | M. Macel | Central Europe | Native |
| Doorweth | The Netherlands | M. Macel | Central Europe | Native |
| Caniac- du-Causse | France | CR 2113 | Mediterranean | Native |
| Saxony-Anhalt† | Germany | PI633142 | Central Europe | Native |
