# Supplementary material for: The Interaction between Root Herbivory and Competitive Ability of Native and Invasive-Range Populations of Brassica nigra
Source: PLoS One. 2015 Oct 30;10(10):e0141857. doi: 10.1371/journal.pone.0141857 (PMC4627727; doi:10.1371/journal.pone.0141857)
Supplement: S2 Table — Species marked by † were obtained from United States Department of Agriculture (USDA) GRIN germplasm collections. (DOC) [file pone.0141857.s006.doc]

# S2 Table

| Species name | Growth form | Accession number  or collector’s name | Source of seed collections |
| --- | --- | --- | --- |
| *Elymus glaucus*† | Grass | W6 32929 | North America |
| *Nassella pulchra*† | Grass | NSL 439946 | North America |
| *Medicago lupulina*† | Forb | PI 577340 | North America |
| *Sonchus oleraceus* | Forb | S.Y. Strauss | North America |
| *Achillea millefolium* | Forb | Rieger Hofmann GmbH | Germany |
