# Supplementary material for: The Interaction between Root Herbivory and Competitive Ability of Native and Invasive-Range Populations of Brassica nigra
Source: PLoS One. 2015 Oct 30;10(10):e0141857. doi: 10.1371/journal.pone.0141857 (PMC4627727; doi:10.1371/journal.pone.0141857)
Supplement: S3 Table — (DOC) [file pone.0141857.s007.doc]

S3 Table

|  | PC1 (41%) | PC2 (24%) | PC3 (21%) | PC4 (14%) |
| --- | --- | --- | --- | --- |
| *E. glaucus* | 0.54 | -0.41 | -0.42 | -0.60 |
| *N. pulchra* | 0.61 | -0.26 | -0.01 | 0.75 |
| *S. oleraceus* | 0.31 | 0.83 | -0.46 | 0.03 |
| *M. lupulina* | 0.48 | 0.26 | 0.78 | -0.30 |
